# Brain network evolution in late preterm to term infants: a near-infrared spectroscopy imaging study

**DOI:** 10.1117/1.NPh.12.3.035016

**Published:** 2025-09-27

**Authors:** Cheng Peng, Fengyu Sun, Lili Liu, Ying Wang, Yang Zhang, Fang Niu, Juan Yang, Yongjia Ji, Qinglan Chen, Xinlin Hou, Yiwei Li

**Affiliations:** a Peking University First Hospital, Children’s Medical Center, Beijing, China; b PKUFH-NINGXIA Women & Children’s Hospital, Department of Neonatology, Ningxia, China; c Geely University of China, School of Business, Chengdu, China; d Sichuan Normal University, Institute of Brain and Psychological Sciences, Chengdu, China

**Keywords:** brain networks, functional near-infrared spectroscopy, preterm, neurodevelopment

## Abstract

**Significance:**

Neonatal brain development plays a crucial role in long-term neurodevelopmental outcomes, particularly in preterm infants.

**Aim:**

We utilized functional near-infrared spectroscopy (fNIRS) to examine the evolution of brain network connectivity in late preterm and term neonates.

**Approach:**

Neonates with a gestational age (GA) between 33 and 41 weeks were included in the study. fNIRS headcaps were placed on the neonates after reaching a stable sleep state. fNIRS data were recorded in continuous-wave mode. Multivariate pattern analysis (MVPA) was conducted to identify distributed patterns of connectivity changes.

**Results:**

Significant developmental changes in brain network connectivity were observed at around 37 weeks of GA, marked by enhanced functional connectivity, particularly within brain network connectivity centered on the parietal lobe (PL). MVPA demonstrated high classification accuracy in distinguishing neonates born before 37 weeks from those born at or after 37 weeks, based on the strength of PL-centered brain connectivity. The accuracy values were as follows: PL = 74.17%, PL-FL = 81.10%, PL-TL = 74.68%, and PL-OL = 67.18%.

**Conclusions:**

These results underscore the critical role of GA in shaping neonatal brain network functional organization and provide valuable insights for early intervention strategies in preterm infants.

## 1 Introduction

The human brain develops from the fetal period through several years after birth. During the fetal stage, the proliferation and migration of neural and glial cells lay the foundation for brain development.^1^ After birth, the brain undergoes progressive maturation, with the neonatal period serving as a critical stage that influences brain function throughout life.^2^ During this stage, the developmental trajectory of the brain may affect susceptibility to lifelong brain-related disorders. Structural variations from the fetal period demonstrate rapid brain growth between birth and up to 2 years of age, particularly in gray matter volume, cortical thickness, and cortical surface area.^3^ Objective and quantitative assessments of neonatal brain function are essential for investigating developmental transition patterns, particularly in preterm infants.^4^

Advancements in perinatal medicine and improvements in neonatal resuscitation and intensive care have led to increased rates of preterm births and successful treatment outcomes.^5^ However, preterm birth remains associated with significant health challenges. Preterm infants, separated prematurely from the maternal environment, are born with underdeveloped organs and face various challenging external conditions. These factors put them at high risk of serious health complications that can hinder early development, particularly those affecting neurological development.^6^^,^^7^ Although survival rates of preterm infants have improved, the incidence of neurological sequelae has not significantly declined. Severe cases can result in long-term neurodevelopmental disabilities, including cerebral palsy, epilepsy, impaired executive function, and sensory impairments.^8^^,^^9^ Consequently, current efforts extend beyond survival to prioritizing quality of life and neurodevelopmental outcomes in preterm infants.

Preterm infants are born during critical stages of brain development. Research has shown that compared with full-term infants, preterm infants at term-equivalent age have significantly reduced volumes of cortical gray matter, deep nuclear gray matter, and regional white matter. Those with substantial reductions in cortical and deep nuclear gray matter volumes are at a higher risk of developing moderate-to-severe neurodevelopmental impairment.^10^^–^^12^ Limited research has investigated the effects of preterm birth on neonatal brain functional connectivity. Existing studies consistently report specific impairments in functional connectivity among brain regions in preterm infants, which are associated with deficits in motor, language, and cognitive development during early childhood and school-age years.^13^^,^^14^ These findings suggest that preterm infants exhibit functional connectivity deficits in brain networks from birth, which may underlie the motor and cognitive impairments observed during childhood development. These findings highlight the need to understand the specific neural vulnerabilities associated with premature birth to better support the cognitive and functional development of this population.

During the early postnatal period, bedside monitoring of key developmental milestones in preterm infants primarily relies on clinical history, observed symptoms, and physical examinations. These assessments evaluate basic motor skills, reflexes, and general physical growth, providing insights into the neurological and developmental statuses of the infants.^15^ It remains unclear whether the development of functional connectivity in the brain networks of preterm infants is characterized by distinct developmental milestones. In addition, do functional connectivity patterns in brain networks differ between infants [gestational age (GA) = 37 to 38 weeks] and infants (GA = 39 to 40 weeks), as defined by the refined “full-term” classification?^16^ Identifying these developmental milestones could not only enhance our understanding of functional connectivity development in the brains of preterm infants but also provide critical insights into early biomarkers for neurodevelopmental disorders, such as autism spectrum disorder.

In our previous studies, we successfully used functional near-infrared spectroscopy (fNIRS) to investigate brain networks in infants with hypoxic-ischemic brain damage (HIBD). Compared with healthy controls, infants with HIBD exhibited reduced functional brain connectivity.^17^ These findings highlight the potential of fNIRS as a screening tool for early detection of neurological disorders. Building on this work, we aimed to expand our research by investigating the developmental trajectory of brain networks during the early postnatal period. Our focus was on understanding how these networks evolve from preterm to term infants and identifying critical milestones in this process. This study aimed to uncover new insights into the complex mechanisms underlying early brain development.

## 2 Materials and Methods

### 2.1 Participants

This study included neonates admitted to the neonatal ward and neonatal intensive care unit of Peking University First Hospital between December 2020 and July 2024. Eligible participants were neonates with a GA between 33 and 41 weeks, assessed within 7 days post-birth. Neonates were grouped based on corrected GA into four 2-week intervals: 33 to $34^{+6}$, 35 to $36^{+6}$, 37 to $38^{+6}$, and 39 to $40^{+6}$ weeks. fNIRS monitoring was performed while neonates were in a quiet resting state within 7 days after birth, with a monitoring duration of 8 min. Neonates were included in the study if they met the following criteria: (1) written informed consent was obtained from their parents; (2) no evidence of brain injury was found based on clinical symptoms, physical examination, and cranial imaging; and (3) no new onset of intracranial hemorrhage. Neonates were excluded if they met any of the following conditions: (1) treatment was discontinued due to social factors, (2) near-infrared spectroscopy data collection could not be completed due to excessive crying or difficulty calming the neonate, or (3) family members declined participation. This approach ensured that brain connectivity differences were not confounded by underlying neuropathological conditions.

### 2.2 Experimental Procedure

Before fNIRS monitoring, neonates were swaddled and placed in a supine position in a crib. After feeding, they were allowed to fall asleep naturally, and an fNIRS headcap was applied once they reached a stable sleep state. Continuous electrocardiogram (ECG) monitoring was performed throughout the procedure, with the monitor set to silent mode. The research team remained bedside to closely observe the neonates. If any changes in vital signs occurred, measurements were immediately discontinued, and appropriate interventions were provided. After completing the procedure, the head cap was removed, and the ECG monitor was reset to its normal alarm settings. No ongoing treatments, including intravenous fluids or antibiotics, were interrupted during the experiment.

### 2.3 fNIRS Data Collection and Analysis

#### 2.3.1 Data collection

**Fig. 1:**
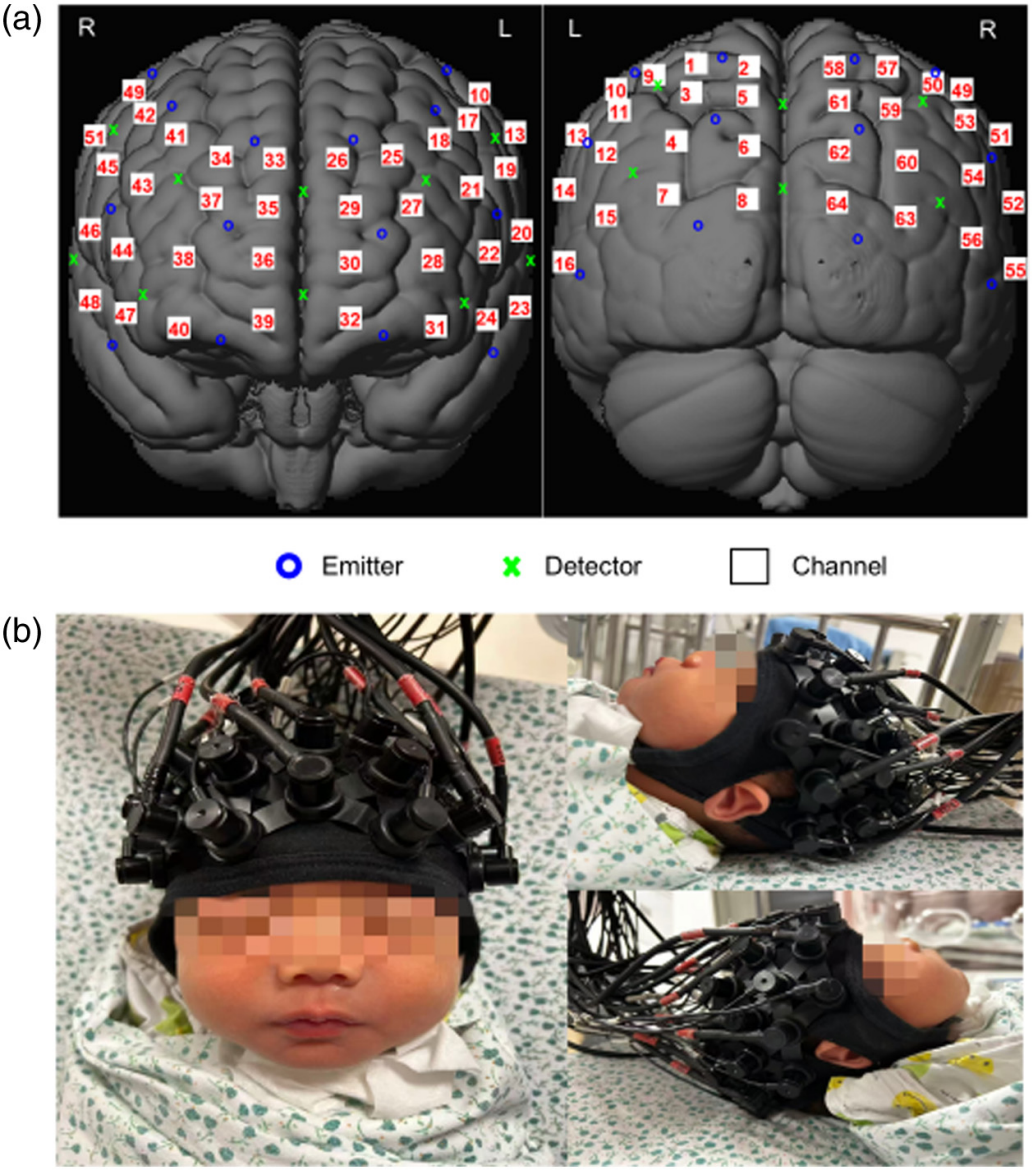
Schematic of functional near-infrared spectroscopy (fNIRS) channel localization on the brain surface. (a) The 24×16 optodes constitute 64 channels. (b) Placement of the neonatal fNIRS cap is illustrated.

fNIRS data were recorded using the NirScan system (Danyang Hui Chuang Medical Equipment, Danyang, China) in continuous-wave mode. The system comprised 24 light emitting diodes (LED) emitters (mean intensity = 5 mW/wavelength) and 16 dual-wavelength detectors (760 and 850 nm) with a data sampling rate of 10 Hz. Optical probes covered the frontal–parietal and temporal–occipital brain regions, forming 64 valid channels [Fig. 1(a)]. The optical probes were secured using two sizes of NIRS-EEG-compatible caps (EASYCAP, Herrsching, Germany): medium with a circumference of 34 cm and small with 32 cm. The distance between each emitter and detector was set to 2.5 cm for the medium cap and 2.3 cm for the small cap. The central Montreal Neurological Institute (MNI) coordinates of each channel’s light source and detector were mapped onto the automated anatomical labeling (AAL) system to establish a correspondence between the fNIRS channels and the cortical structures.^18^ The neonatal testing setup is shown in Fig. 1(b), and the cortical locations corresponding to the 64 channels are listed in Table S1 in the Supplementary Material.

#### 2.3.2 Univariate analysis

Resting-state connectivity among brain regions was estimated 3 min before each testing session using a sample size of 1800.^19^ The Homer2 toolbox was used for quantitative analysis of fNIRS signals and to calculate the concentration changes of oxyhemoglobin (Δ[HbO]).^20^ Noisy channels were identified and removed from the dataset using the Homer2 function hmrPruneChannels (SNRthres = 2). Optical density values were computed from the optical intensity signals, and motion artifacts were corrected using a time-derivative distribution repair algorithm.^21^ A band-pass filter (0.01 to 0.2 Hz) was applied to eliminate physiological noise and baseline drift. Changes in Δ[HbO] and deoxyhemoglobin (Δ[Hb]) concentrations were derived from optical density signals using the modified Beer–Lambert law (differential pathlength factor = 5).^22^^–^^24^ Statistical analyses focused on Δ[HbO] due to its superior sensitivity in evaluating functional activity across different conditions.^25^ Pearson correlation coefficients were computed between the time series of each pair of fNIRS channels to estimate spontaneous functional connectivity, generating a matrix of correlation coefficients (r-values). These r-values were transformed into Fisher’s z-scores to approximate a normal distribution for statistical analysis.^26^ Regions of interest (ROIs) were defined based on specific AAL (Table S1 in the Supplementary Material). The corresponding channels for each ROI were as follows: (1) frontal lobe (FL): channels 18, 21, 22, 24 to 44, and 47; (2) parietal lobe (PL): channels 1 to 3, 5, 9 to 14, 17, 19, 20, 42, 45, 46, 49 to 54, 57 to 59, and 61; (3) temporal lobe (TL): channels 15, 16, 23, 48, 55, and 56; and (4) occipital lobe (OL): channels 4, 6 to 8, 60, and 62 to 64.

Complex network analysis was conducted using the GRETNA toolbox.^27^ Brain network-related metrics primarily consisted of global indices, which included small-world parameters, such as the clustering coefficient, characteristic path length, and sigma. As different metrics can be obtained at various thresholds, the average values were calculated at a sparsity level of 35% to 40% for further analysis.

#### 2.3.3 Multivariate pattern analysis

Multivariate pattern analysis (MVPA) was conducted to identify distributed patterns of connectivity changes.^28^ The analysis was designed to predict group labels (33 to $36^{+6}$ and 37 to $40^{+6}$ weeks) for each neonate based on the strength of their brain connections. A total of 11 MVPA analyses were performed. First, to assess whether whole-brain functional connectivity could distinguish group classifications, 2016 pairs of functional connectivity strengths (z-scores) were used as input features. Second, to examine the differences in brain network development across the four ROIs, functional connections within and between the FL, PL, TL, and OL were selected as features for analysis.

A linear support vector machine was implemented using MVPA light to analyze the functional connectivity strength values of all neonates.^29^ The classifier was trained and tested using a fivefold cross-validation procedure. Group classifications were assigned as follows: neonates aged 33 to $36^{+6}$ weeks were labeled as −1, and those aged 37 to $40^{+6}$ weeks were labeled as 1.

### 2.4 Statistical Analysis

Statistical analyses were conducted using RStudio software (v2021.09.0, RStudio, Boston, Massachusetts, United States). Descriptive statistics are presented as “mean ± standard error (SE).” The significance level was set at 0.05, and effect sizes for analysis of variance (ANOVA) were reported using $\eta_{p}^{2}$. Multiple comparisons were adjusted using the false discovery rate (FDR) method.^30^

To further characterize the developmental trajectory, brain-network-related metrics for neonates aged 33 to 40 weeks GA were modeled using the following sigmoid function.^31^ In this formulation, x represents GA (independent variable) and y represents the predicted magnitude of brain network-related metrics (dependent variable). This sigmoid model addresses the nonlinear least-squares fitting problem and yields several key parameters, including the central point (Xc), corresponding to the abscissa value at the function’s inflection, and the slope parameter (b), which quantifies the steepness of the curve at Xc

$$y(x)=\frac{y_{\min}+y_{\min}\times e^{\frac{x-x_{c}}{b}}}{1+e^{\frac{x-x_{c}}{b}}}.$$

For the multivariate statistical analysis, a conservative permutation test was employed, as the binomial test is known to be overly optimistic for MVPA cross-validation schemes.^32^ In the permutation test, the null distribution of the classification accuracy was generated by randomly permuting group labels 10,000 times. Each iteration included a complete run of the cross-validation scheme, incorporating a fivefold cross-validation.^33^

## 3 Results

The demographic and clinical characteristics of the groups categorized by post-menstrual age (PMA) are shown in Table 1. Gender distribution was similar across groups ($\chi^{2}=0.797$, p=0.850). Significant differences were observed in PMA, GA, HC, Cesarean section, and birth weight (BW) among the groups (all p<0.001), indicating developmental variations based on PMA classification. There were no significant differences among the four groups with respect to gender distribution, Apgar scores at 1 and 5 min, hematocrit, aEEG completion rate, and the proportion of normal aEEG patterns (all p>0.05). Statistical analyses included one-way ANOVA and Pearson’s chi-square test.

**Table 1:** Demographic and clinical characteristics of groups categorized by post-menstrual age [mean ± standard deviation (SD)].

Items	33 to $34^{+6}$	35 to $36^{+6}$	37 to $38^{+6}$	39 to $40^{+6}$	Statistics^a^
	(n=14)	(n=16)	(n=16)	(n=22)	
Gender (male/female)	7/7	10/6	9/7	12/10	$\chi^{2}=0.797$, p=0.850
PMA (weeks)	34.08 ± 0.66	35.75 ± 0.71	38.19 ± 0.61	40.17 ± 0.40	F(3,64)=361.685, p<0.001
GA (weeks)	33.34 ± 1.01	35.16 ± 0.98	37.83 ± 0.59	39.59 ± 0.64	F(3,64)=205.051, p<0.001
BW (g)	1983.57 ± 365.85	2243.44 ± 181.68	2983.13 ± 437.54	3461.82 ± 335.28	F(3,64)=69.540, p<0.001
Cesarean section (n)	13	13	7	5	$\chi^{2}=21.46$, p<0.001
HC (cm)	30.86 ± 1.68	31.69 ± 1.09	33.03 ± 1.33	33.91 ± 0.93	F(3,64)=24.699, p<0.001
APGAR score 1 min	9.64 ± 0.63	9.75 ± 0.58	9.75 ± 0.58	9.77 ± 0.53	F(3,64)=0.159, p=0.923
APGAR score 5 min	10.00 ± 0.00	9.94 ± 0.25	10.00 ± 0.00	9.91 ± 0.29	F(3,64)=0.867, p=0.463
Hematocrit (%)	46.56 ± 4.09	46.46 ± 4.98	49.68 ± 5.47	45.90 ± 4.83	F(3,64)=2.080, p=0.112
aEEG completed (n)	14	14	14	21	$\chi^{2}=2.61$, p=0.456
Normal aEEG pattern (n)	13	14	13	19	$\chi^{2}=1.33$, p=0.721

Abbreviations: PMA, post-menstrual age (the age of an infant calculated from the last menstrual period); GA, gestational age (the age of the fetus or newborn from conception); BW, birth weight (infant weight at birth); HC, head circumference (measurement of the infant’s head size); APGAR, appearance, pulse, grimace, activity, and respiration; aEEG, amplitude-integrated electroencephalography.

a One-way ANOVA or Pearson’s chi-squared test.

### 3.1 Univariate Analyses

**Fig. 2:**
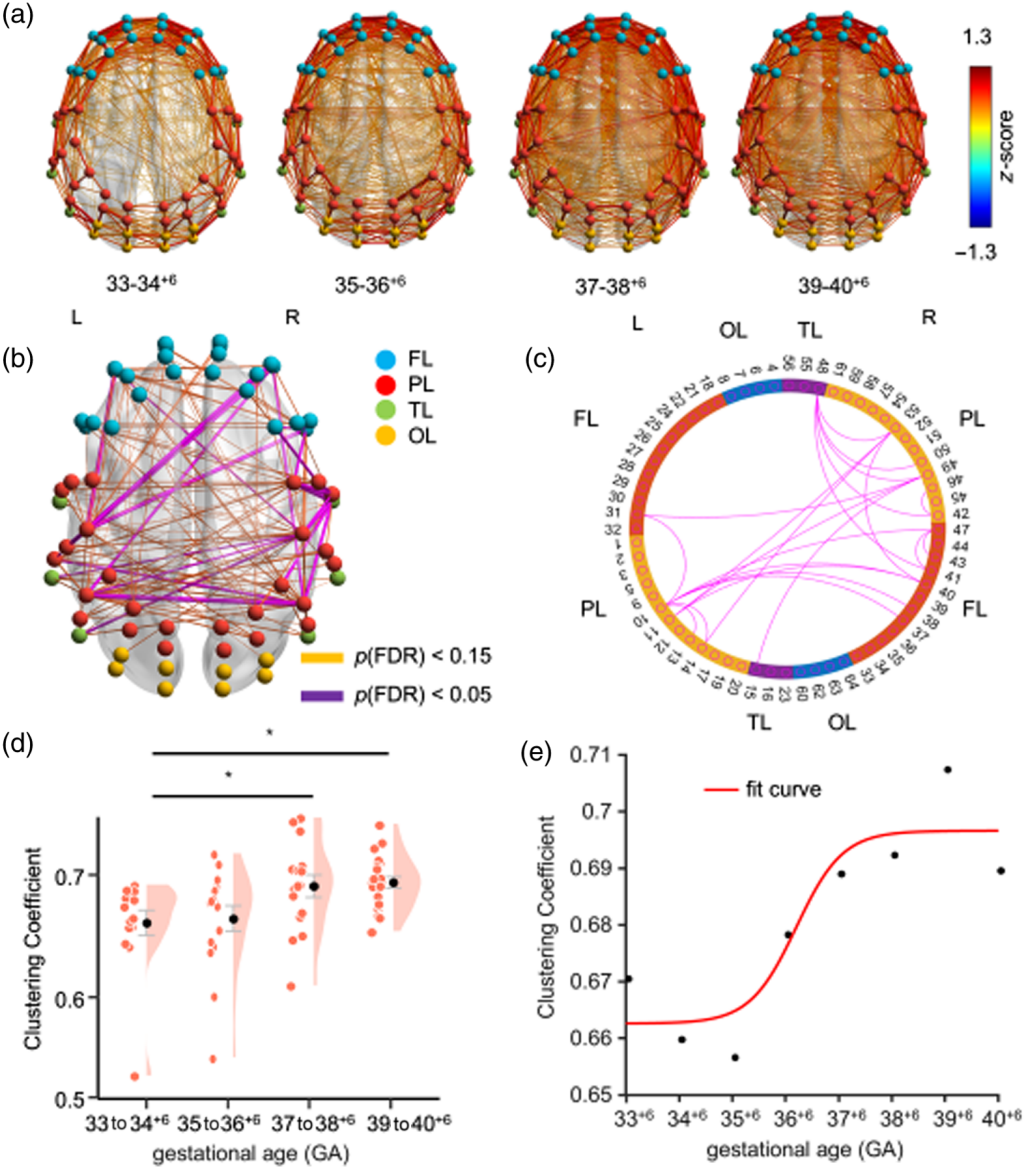
Results of brain network connectivity. (a) Mean values of brain network connectivity across four groups of neonates. (b) and (c) Results of one-way ANOVA analysis. (d) Network analysis revealed significant differences in clustering coefficients at gestational age (GA) of 33 to $34^{+6}$ weeks, 35 to $36^{+6}$ weeks, 37 to $38^{+6}$ weeks, and 39 to $40^{+6}$ weeks. (e) Clustering coefficients fitted non-linearly using a sigmoid function. Significance levels are indicated as follows: *P(FDR)<0.05. Abbreviations: GA, gestational age; FL, frontal lobe; PL, parietal lobe; OL, occipital lobe; TL, temporal lobe; FDR, false discovery rate.

To assess the changes in neonates across the four GA groups, a one-way ANOVA was conducted on the z-scores for the groups (33 to $34^{+6}$, 35 to $36^{+6}$, 37 to $38^{+6}$, and 39 to $40^{+6}$ weeks). The analysis revealed significant differences in 25 pairs of brain connections among the GA groups [F(3,64)≥6.571, $\eta_{p}^{2}=0.121$, p(FDR)<0.05; Figs. 2(b) and 2(c)], with the brain connection strengths in the groups of 37 weeks and above being significantly higher than those below 37 weeks [Fig. 2(a)]. These significant differences were primarily concentrated within the bilateral hemispheres.

Further analysis indicated that 3 pairs were within the left hemisphere (LPL: 2 pairs; LPL-LPFC: 1 pair), 12 pairs were within the right hemisphere (RPFC: 2 pairs; RPL: 3 pairs; RPFC-RPL: 2 pairs; RPFC-RTL: 1 pair; RPL-RTL: 4 pairs), and 10 pairs were interhemispheric functional connections (RPFC-LPL: 5 pairs; RPL-LPFC: 1 pair; RPL-LPL: 3 pairs; RPL-LTL: 1 pair). Specifically, connections within the PL and between the PL and other ROIs accounted for 88% of the identified pairs.

In the brain network analysis, one-way ANOVA results for the clustering coefficient across the four neonatal groups revealed significant differences in the clustering coefficients [F(3,64)=4.244, p(FDR)=0.026, $\eta_{p}^{2}=0.166$; Fig. 2(d)]. A simple effects analysis indicated a significant decrease in the clustering coefficient from the 37 to $38^{+6}$ (0.692±0.009) and 39 to $40^{+6}$ weeks groups (0.695±0.007) to the 33 to $34^{+6}$ weeks group (0.662±0.009) (37 to $38^{+6}$ versus 33 to $34^{+6}$: t(64)=2.368, p=0.042; 39 to $40^{+6}$ versus 33 to $34^{+6}$: t(64)=2.802, p =0.034). No significant differences were observed in characteristic path length or sigma (Fs<1). Nonlinear fitting results revealed a sharp increase in clustering coefficients around 37 weeks of GA [Fig. 2(e)], with the central point estimated at Xc=36.2 (95% CI: 34.74 to 37.66), where Xc denotes the inflection point of the sigmoid function.

### 3.2 Multivariate Pattern Analysis

**Fig. 3:**
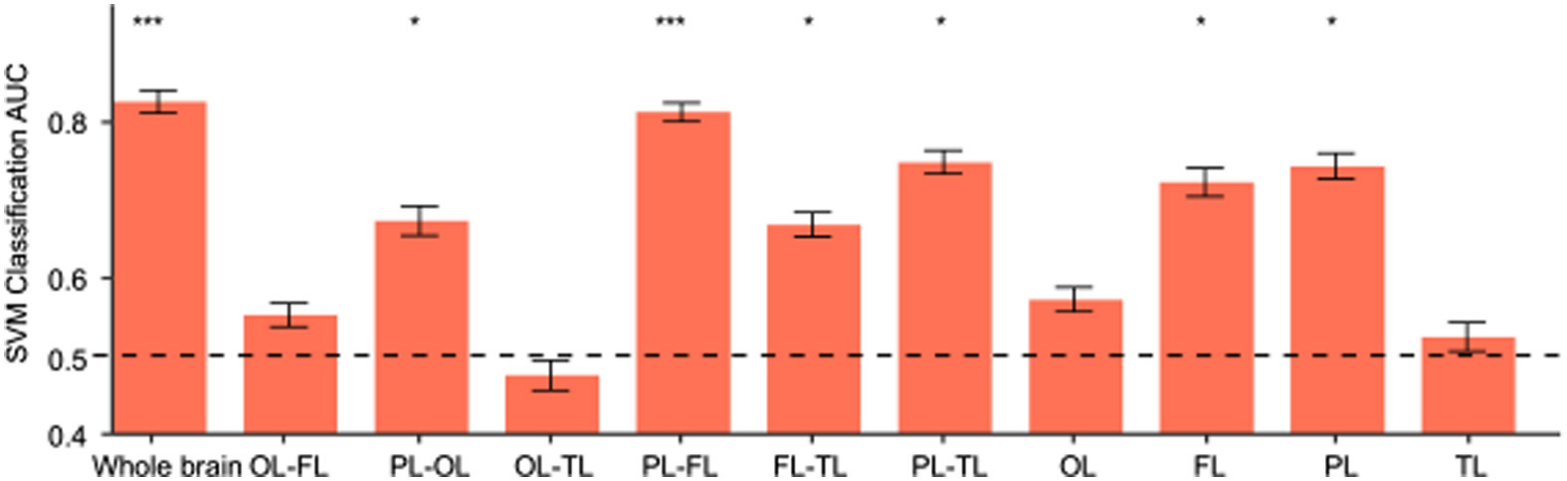
Results of MVPA. MVPA analysis of brain network connectivity across the whole brain, as well as within and between the four ROIs (FL, PL, OL, and TL). Significance levels are indicated as follows: *P<0.05, ***P<0.001, false discovery rate (FDR)-corrected for 11 permutation tests. Abbreviations: FL, frontal lobe; PL, parietal lobe; OL, occipital lobe; TL, temporal lobe.

These results suggest that 37 weeks is a critical period for changes in brain connectivity. Consequently, we regrouped the neonates into two categories: 33 to $36^{+6}$ weeks and 37 to $40^{+6}$ weeks for MVPA analysis. The analysis revealed that whole-brain connectivity could predict group labels with high accuracy [area under the curve (AUC) = 82.38%, p(FDR)<0.001, chance-level accuracy = 50%; Fig. 3].

In addition, MVPA was conducted on the brain network connections within and between the four ROIs. The results demonstrated that network connectivity within the PL and FL accurately predicted group labels [PL: AUC = 74.17%, p(FDR)=0.0133; FL: AUC = 72.09%, p(FDR)=0.020; Fig. 3]. Connectivity between the PL and other ROIs, as well as between the FL and TL, also achieved high predictive accuracy [PL-OL: AUC = 67.18%, p(FDR)=0.045; PL-FL: AUC = 81.10%, p(FDR)<0.001; PL-TL: AUC = 74.68%, p(FDR)=0.010; FL-TL: AUC = 66.69%, p(FDR)=0.045; Fig. 3].

## 4 Discussion and Conclusion

This study investigated the key changes in neonatal brain network development by examining resting-state brain network connectivity. The findings revealed a significant turning point at 37 weeks GA, marking the onset of rapid brain network development in neonates. These differences were observed both within and between hemispheres, particularly in brain networks associated with the PL. This suggests that neonates born at or near term exhibited more advanced and organized neural network configurations. Further analysis of network properties revealed that the clustering coefficient, which reflects local brain network integration, was significantly lower in the preterm group (33 to $34^{+6}$ weeks) than in those born at 37 weeks and beyond. This indicates a more mature network organization in the latter cohort. In addition, MVPA demonstrated that brain connectivity patterns, particularly those related to the PL, could reliably predict GA group classification. These results highlight the potential of fNIRS as a noninvasive biomarker for assessing early brain maturation and underscore the crucial role of the PL in early development. These findings emphasize the critical role of GA in shaping the functional organization of the neonatal brain, with substantial neural maturation occurring at ∼37 weeks of age. These findings may enhance our understanding of the neurodevelopmental trajectory of preterm infants and may inform early intervention strategies.

Our findings reveal that functional connectivity and clustering coefficients in the brain networks of preterm infants undergo significant changes around 37 weeks of gestation, whereas no comparable changes are observed between 37 and 40 weeks. These findings support the conventional perspective that 37 weeks of gestation represents a critical threshold for fetal maturity, with infants born between 37 and 41 weeks typically regarded as healthy and classified as a homogeneous cohort. However, recent studies have identified significant variations in both physical and cognitive development within the 37- to 41-week window. Research indicates that neonates born at 37 to 38 weeks’ GA face higher risks of neonatal mortality and exhibit increased morbidity in respiratory, neurological, and endocrine health compared with those born at 39 to 41 weeks.^34^^–^^40^ In addition, studies suggest that neonates born before 38 weeks of GA tend to show poorer developmental and academic outcomes during school years than those born at or after 39 weeks.^41^^–^^47^ Consequently, the definition of full-term pregnancy has been narrowed to a 1-week window beginning at 39 weeks;^16^ nonmedically indicated deliveries between 37 and 38 week GA are discouraged (ACOG Committee Opinion No. 765, 2019). Although accumulating evidence highlights the drawbacks of the traditional 37-week threshold, our findings contribute to the limited body of research suggesting that the neonatal brain network reaches a developmental milestone at 37 weeks GA.

This fNIRS study demonstrated that functional connectivity between the PL region and other brain areas increased with advancing GA, indicating enhanced coordination and integration of neural networks with brain maturity. The PL plays a critical role in integrating sensory and motor signals to support movement planning, higher-order motor control, action-perception linkage, and complex motor coordination.^48^^,^^49^ In preterm infants, reduced functional connectivity between the PL and other brain regions, such as the frontotemporal cortex, is linked to abnormal general movements in early infancy and predicts poorer cognitive and motor outcomes at two years of age.^50^ In addition, structural brain studies in preterm infants indicate that greater white matter injury volumes in the PL are associated with adverse motor outcomes.^51^ Collectively, these studies underscore the critical role of the PL in early brain development, particularly in integrating sensory and motor functions. Consistent with prior research, our findings demonstrate that internal connectivity within the right prefrontal cortex of preterm infants is similarly impaired, impacting the development of higher-order cognitive and executive functions.^52^ Impaired connectivity in the right prefrontal cortex is linked to subsequent cognitive and executive function deficits in preterm infants. Moreover, some studies report that these connectivity abnormalities persist into adulthood, correlating with declines in cognitive and language comprehension abilities.^53^

The clustering coefficient quantifies the extent to which brain regions form tightly connected clusters, facilitating specialized information processing.^54^ In preterm infants, both structural and functional brain networks exhibit reduced clustering coefficients compared with term-born peers at term-equivalent age, indicating less efficient local connectivity and impaired network segregation.^55^^,^^56^ However, unlike previous studies, our findings did not consistently identify other network alterations, such as reduced local efficiency or modularity, particularly in regions critical for cognitive, linguistic, and motor functions.^55^

Beyond network property analysis, our findings are consistent with previous fNIRS studies in emphasizing GA-related influences on resting-state network development. Although Arimitsu et al.^57^ reported that higher-GA preterm infants (≥30 weeks) demonstrated stronger inter-area connectivity compared with both lower-GA preterm and term infants, our results showed a different but complementary pattern: connectivity increased with GA up to 37 weeks and then plateaued, with term infants exhibiting levels comparable to preterm infants. This suggests that GA primarily determines baseline connectivity in the early neonatal period, reflecting greater structural and functional maturity at higher GA. The observation that developing preterm infants sometimes show higher connectivity than term infants may represent a compensatory enhancement during the catch-up process of network maturation. However, this catch-up occurs more slowly in infants born at very low GA, which could account for their higher risk of potential neurodevelopmental impairment.

The clinical applicability of fNIRS in neonatal brain monitoring is further supported by studies in pathological populations. Kebaya et al.^58^ integrated three-dimensional cranial ultrasound with multichannel fNIRS to monitor germinal matrix–intraventricular hemorrhage (IVH) in preterm neonates, revealing that increased ventricular volumes were significantly associated with reduced spontaneous functional connectivity magnitudes. This supports the notion that structural disruptions in early brain injury can be sensitively reflected in functional network alterations. Moreover, the research team evaluated resting-state functional connectivity (RSFC) in very preterm neonates with IVH using fNIRS compared with fMRI. fNIRS maps showed good correspondence with fMRI.^59^ Findings suggest fNIRS is a feasible bedside tool for assessing cortical RSFC and may serve as a biomarker for IVH severity.

Our findings suggest that brain network connectivity can predict the maturity of an infant’s physiological development, with significant implications for early detection, diagnosis, and intervention in preterm infants. The timely identification of at-risk infants enables early targeted interventions that can mitigate the severity of cognitive and neurological impairments.^60^ This not only improves the quality of life for affected children but also reduces the long-term costs associated with rehabilitation, special education, and healthcare. Investing in early screening and interventions improves health outcomes and can lead to substantial long-term cost savings. To fully harness the potential of early neurodevelopmental prediction in preterm infants, future research should focus on developing large-scale longitudinal databases. These databases are essential for establishing normative reference ranges for early brain network biomarkers across various GAs in preterm neonates. In addition, defining specific reference values for infants with abnormal neonatal conditions, including central nervous system infections, intraventricular hemorrhage, perinatal brain injury, hypoxic-ischemic encephalopathy, bilirubin-induced neurological dysfunction, and hypoglycemic encephalopathy, is essential.

Despite these implications, this study has some limitations. First, although fNIRS is a valuable tool, its measurement accuracy may be affected by factors such as neonatal movement. Second, due to the observational nature of the study, a causal relationship between GA and cerebral development cannot be established. Third, as the study focused on neonates within the first 7 days of birth, the results may not be applicable to older neonates or those with comorbidities. Larger multicenter studies are necessary to validate these findings. In addition, although fNIRS has the advantages of being non-invasive, easy to administer, and repeatable and has therefore been widely applied in neonatal research,^61^^,^^62^ it is still necessary to consider the impact of the immature development of the neonatal scalp and skull on fNIRS signal acquisition. For instance, because neonates have relatively thin scalp and skull tissues, the extracranial contribution to the signal remains relatively low. Finally, our fNIRS-based brain network functional connectivity results further suggest that 37 weeks GA may represent a critical period in neurodevelopment. However, this interpretation requires validation from additional modalities, such as EEG and structural MRI. Notably, related EEG research employing an oddball paradigm to examine the developmental trajectory of emotional speech discrimination in neonates reported a marked developmental shift at GA 37 weeks, indicating heightened perceptual sensitivity to emotional vocal expressions. Infants younger than 37 weeks GA were able to discriminate emotional prosody, whereas those older than 37 weeks GA exhibited enhanced discrimination abilities.^63^ Future research may benefit from simultaneously applying EEG and fNIRS to the same cohort of neonates across different PMA stages, enabling longitudinal observation of the brain maturation process.

This study highlights the pivotal influence of GA on neonatal brain network development, with substantial maturation occurring around 37 weeks of GA. Brain networks centered on the PL were shown to accurately predict neurodevelopmental status in newborns. Early detection and timely intervention are crucial for enhancing long-term outcomes and reducing healthcare costs associated with neurodevelopmental disorders. Further research and the establishment of comprehensive databases are essential to improve diagnostic criteria and intervention strategies for preterm infants.

## Supplementary Material

10.1117/1.NPh.12.3.035016.s01

## Data Availability

The preprocessed data and scripts are available at https://osf.io/9jnfd/, and the raw data can be provided upon reasonable request, subject to approval from Peking University First Hospital.
