# Supplementary material for: Brain network evolution in late preterm to term infants: a near-infrared spectroscopy imaging study
Source: Neurophotonics. 2025 Sep 27;12(3):035016. doi: 10.1117/1.NPh.12.3.035016 (PMC12476264; doi:10.1117/1.NPh.12.3.035016)
Supplement: Supplementary file 1 [file NPh_012_035016_SD001.docx]

**Table S1 Spatial registration for near-infrared spectroscopy (NIRS) channels using automated anatomical labeling (AAL)**

| CHANNEL | AAL | X (MINI) | Y (MINI) | Z (MINI) |
| --- | --- | --- | --- | --- |
| CH1 (S1-D1) | **Left superior parietal gyrus** | **−29.67** | **−58.67** | **70** |
| CH2 (S1-D15) | **Left superior parietal gyrus** | **−13** | **−68** | **68.67** |
| CH3 (S2-D1) | **Left superior parietal gyrus** | **−31.33** | **−69.33** | **60.67** |
| CH4 (S2-D2) | **Left angular gyrus** | **−35.67** | **−81.67** | **45.67** |
| CH5 (S2-D15) | **Left superior parietal gyrus** | **−13.67** | **−78.33** | **59.67** |
| CH6 (S2-D16) | **Left superior occipital gyrus** | **−13.33** | **−88.33** | **44.67** |
| CH7 (S3-D2) | **Left middle occipital gyrus** | **−38.33** | **−88.67** | **28.67** |
| CH8 (S3-D16) | **Left superior occipital gyrus** | **−13.67** | **−98.67** | **28.33** |
| CH9 (S4-D1) | **Left superior parietal gyrus** | **−41.67** | **−42.33** | **66.33** |
| CH10 (S4-D3) | **Left postcentral gyrus** | **−52.67** | **−19.33** | **59.67** |
| CH11 (S5-D1) | **Left angular gyrus** | **−53** | **−52** | **55** |
| CH12 (S5-D2) | **Left angular gyrus** | **−56** | **−63.67** | **41.67** |
| CH13 (S5-D3) | **Left supramarginal gyrus** | **−62.67** | **−28** | **47.33** |
| CH14 (S5-D4) | **Left supramarginal gyrus** | **−68.67** | **−34** | **29** |
| CH15 (S6-D2) | **Left middle occipital gyrus** | **−56.33** | **−72** | **22.33** |
| CH16 (S6-D4) | **Left middle temporal gyrus** | **−70** | **−43.33** | **7.33** |
| CH17 (S7-D3) | **Left precentral gyrus** | **−50** | **5.33** | **52.33** |
| CH18 (S7-D5) | **Left middle frontal gyrus** | **−40.33** | **31.33** | **45.67** |
| CH19 (S8-D3) | **Left precentral gyrus** | **−60.33** | **4.33** | **36.67** |
| CH20 (S8-D4) | **Left postcentral gyrus** | **−65** | **0.33** | **17.67** |
| CH21 (S8-D5) | **Left middle frontal gyrus** | **−49.67** | **33.67** | **30.67** |
| CH22 (S8-D6) | **Left inferior frontal gyrus** | **−55** | **34.67** | **10.67** |
| CH23 (S9-D4) | **Left superior temporal gyrus** | **−66** | **−5.33** | **−5.33** |
| CH24 (S9-D6) | **Left lateral orbitofrontal gyrus** | **−53** | **32** | **−9** |
| CH25 (S10-D5) | **Left middle frontal gyrus** | **−26.67** | **47.67** | **41.67** |
| CH26 (S10-D7) | **Left superior frontal gyrus** | **−9.67** | **59** | **40.67** |
| CH27 (S11-D5) | **Left middle frontal gyrus** | **−32.67** | **57** | **26.67** |
| CH28 (S11-D6) | **Left middle frontal gyrus** | **−38.67** | **60.67** | **8.67** |
| CH29 (S11-D7) | **Left superior frontal gyrus** | **−13.67** | **66.67** | **25.67** |
| CH30 (S11-D8) | **Left superior frontal gyrus** | **−13.67** | **73** | **8.67** |
| CH31 (S12-D6) | **Left lateral orbitofrontal gyrus** | **−38.67** | **60** | **−11** |
| CH32 (S12-D8) | **Left superior frontal gyrus** | **−13.33** | **71** | **−9.33** |
| CH33 (S13-D7) | **Left superior frontal gyrus** | **9.67** | **58.67** | **40.33** |
| CH34 (S13-D9) | **Right middle frontal gyrus** | **26.67** | **49.67** | **42.67** |
| CH35 (S14-D7) | **Right middle frontal gyrus** | **12.67** | **68** | **26.33** |
| CH36 (S14-D8) | **Right middle frontal gyrus** | **13.33** | **74** | **9.33** |
| CH37 (S14-D9) | **Right middle frontal gyrus** | **31.67** | **57.67** | **28.67** |
| CH38 (S14-D10) | **Right middle frontal gyrus** | **38.67** | **63** | **11** |
| CH39 (S15-D8) | **Right middle frontal gyrus** | **13.33** | **72** | **−9.67** |
| CH40 (S15-D10) | **Right lateral orbitofrontal gyrus** | **39.67** | **62** | **−12** |
| CH41 (S16-D9) | **Right middle frontal gyrus** | **40.67** | **31.67** | **48** |
| CH42 (S16-D11) | **Right precentral gyrus** | **50** | **4** | **54.67** |
| CH43 (S17-D9) | **Right middle frontal gyrus** | **51.67** | **33.67** | **32.67** |
| CH44 (S17-D10) | **Right inferior frontal gyrus** | **58** | **34.67** | **12.67** |
| CH45 (S17-D11) | **Right precentral gyrus** | **62.33** | **4.33** | **38.33** |
| CH46 (S17-D12) | **Right postcentral gyrus** | **69** | **−1.67** | **18.33** |
| CH47 (S18-D10) | **Right inferior frontal gyrus** | **56** | **30.67** | **−7.67** |
| CH48 (S18-D12) | **Right superior temporal gyrus** | **69** | **−5** | **−4** |
| CH49 (S19-D11) | **Right supramarginal gyrus** | **54.33** | **−21.33** | **60.67** |
| CH50 (S19-D13) | **Right angular gyrus** | **44** | **−47** | **64** |
| CH51 (S20-D11) | **Right supramarginal gyrus** | **66** | **−30** | **48** |
| CH52 (S20-D12) | **Right supramarginal gyrus** | **70** | **−36.33** | **26.33** |
| CH53 (S20-D13) | **Right angular gyrus** | **55** | **−56.33** | **52.33** |
| CH54 (S20-D14) | **Right angular gyrus** | **57** | **−66.67** | **34.67** |
| CH55 (S21-D12) | **Right middle temporal gyrus** | **71.67** | **−43.33** | **6.67** |
| CH56 (S21-D14) | **Right middle occipital gyrus** | **57** | **−72.33** | **15.33** |
| CH57 (S22-D13) | **Right superior parietal gyrus** | **31** | **−60.67** | **69** |
| CH58 (S22-D15) | **Right superior parietal gyrus** | **14** | **−68** | **69** |
| CH59 (S23-D13) | **Right angular gyrus** | **31.67** | **−73.67** | **55.67** |
| CH60 (S23-D14) | **Right middle occipital gyrus** | **36.33** | **−84.33** | **39.33** |
| CH61 (S23-D15) | **Right superior parietal gyrus** | **15.33** | **−79.33** | **57.67** |
| CH62 (S23-D16) | **Right superior occipital gyrus** | **15.33** | **−90** | **42.33** |
| CH63 (S24-D14) | **Right middle occipital gyrus** | **35.67** | **−92** | **21.67** |
| CH64 (S24-D16) | **Right superior occipital gyrus** | **14.67** | **−98.67** | **26.33** |
